# Zero-profile versus cage-plate interbody fusion system in anterior cervical discectomy and fusion for the treatment of multilevel cervical spondylosis: A protocol of systematic review and meta-analysis

**DOI:** 10.1097/MD.0000000000022026

**Published:** 2020-08-28

**Authors:** Ning Li, Rui Wang, Wei Teng, Jinhua Yu

**Affiliations:** Department of Orthopaedic, Qingdao Central Hospital, Shibei District, Qingdao, Shandong Province, China.

**Keywords:** anterior cervical discectomy and fusion, multilevel cervical spondylosis, protocol, systematic review, Zero-P profile

## Abstract

**Background:**

The objective of this meta-analysis was to summarize and identify the available evidence from these studies to estimate which device was better for multilevel cervical spondylotic myelopathy (MCSM). And provides clinicians with evidence on which to base their clinical decision making.

**Methods:**

This review will include all studies comparing the new Zero-profile versus cage-plate interbody fusion system in anterior cervical discectomy and fusion (ACDF) for the treatment of MCSM. The search strategy will be performed in 9 databases. We will not establish any limitations to language and publication status, published from inception to the July, 2020. Two reviewers will screen, select studies, extract data, and assess quality independently. Outcome is operative time, blood loss, clinical function outcome, radiologic outcomes, and complications. The methodological quality including the risk of bias of the included studies will be evaluated. We will carry out statistical analysis using RevMan 5.3 software.

**Results:**

This study will summarize current evidence to assess the efficacy and safety of Zero-profile versus cage-plate interbody fusion system in ACDF for the treatment of MCSM.

**Conclusion:**

The findings of this study will provide helpful evidence for the clinician, and will promote further studies, as well as comparing the 2 devices in ACDF for MCSM

**Registration number:**

INPLASY202070095 (DOI number: 10.37766/inplasy2020.7.0095).

## 1 Introduction

Cervical spondylotic myelopathy (CSM) is the most common cause of spinal cord dysfunction. Surgical treatment is indicated when conservative therapy fails or when the symptoms worsen.^[1,2]^ In 1958, Cloward, Smith, and Robinson first reported that anterior cervical operation is a safe and effective method for the treatment of degenerative cervical spondylosis. Anterior cervical discectomy and fusion (ACDF) is still performed in most cases and is the gold standard for the treatment of cervical spondylosis.^[3–5]^ Anterior cage-plate construct is commonly used in ACDF in order to enhance segmental stability improve cervical sagittal alignment, reduce graft extrusion and subsidence, and increase fusion rates. These techniques have their own benefits as well as potential drawbacks and adverse effects. The most often mentioned shortcomings of these techniques are the breakage or loosening of plate and screws, trachea-esophageal injury, neurovascular injury, and postoperative dysphagia have caused concerns.^[6,7]^ Many studies have reported that an anterior plate with a lower, smoother profile may reduce the incidence of dysphagia after ACDF.^[8,9]^ To reduce these complications, the zero-profile spacer has been introduced and applied for ACDF. The device can be implanted into the intervertebral space entirely, providing adequate stability and avoiding implant contact with the prevertebral soft tissue.

Recently, several studies have compared the clinical and radiologic outcomes of zero-profile spacer and cage-plate construct in ACDF for treating multilevel CSM (MCSM). To the best of our knowledge, there is no meta-analysis comparing the 2 devices in ACDF for MCSM. Consequently, the objective of this meta-analysis was to summarize and identify the available evidence from these studies to estimate which device was better for MCSM. And provides clinicians with evidence on which to base their clinical decision making.

## 2 Methods

### 2.1 Study registry

The protocol was registered on the International Platform of Registered Systematic Review and Meta-analysis Protocols (INPLASY 202078431). The preferred reporting items for systematic review and meta-analysis protocols (PRISMA) will serve as guidelines for reporting present review protocol and subsequent formal paper.^[10]^

### 2.2 Eligibility criteria for including studies

#### 2.2.1 Types of studies

We will include all studies comparing the new Zero-profile versus cage-plate interbody fusion system in ACDF for the treatment of MCSM, including observational study and randomized clinical trials (RCT). Any other types of studies, such as animal studies, case reports, case series, and review will all be excluded.

#### 2.2.2 Types of interventions

##### 2.2.2.1 Experimental group

All patients in the experimental group received Zero-profile interbody fusion for their treatment in this study.

##### 2.2.2.2 Control group

The participants in the control group could receive cage-plate interbody fusion system for their treatment in this study.

#### 2.2.3 Types of patients

All patients with MCSM undergoing ACDF involving ≥2 levels; the study included a comparative design (zero-profile vs cage-plate); follow-up of at least 12 months will all be considered for inclusion. Studies that provided no information about complications and had no specific data on the clinical effect were excluded.

#### 2.2.4 Types of outcome measurements

##### 2.2.4.1 Primary outcomes

Intraoperative time and intraoperative blood loss are important objective bases for the evaluation of operational injury. Therefore, they can have objective through comparison the evaluation of operation trauma.

##### 2.2.4.2 Secondary outcomes

Secondary outcomes mainly involve include decrease of Japanese Orthopaedic Association (JOA) score, radiographic outcome, dysphagia, and other related complications.

### 2.3 Literature sources and search

We will perform literature searches using the following electronic bibliographic databases from their inception onwards to the July, 2020: MEDLINE, Springer, Web of Science, PubMed, EMBASE, the Cochrane Central Register of Controlled Trials, Evidence Based Medicine Reviews, VIP, and CNKI. We will not establish any limitations to language and publication status. The following electronic databases were searched from their inception dates through July 2020: The search was conducted by using the combination of the following terms: “Zero-p” OR “Zero-profile” OR “Zero profile” OR “Stand-alone” OR “anchored spacer” OR “anchored cage” OR “anchored fusion” OR “no-profile” AND “cervical.”

### 2.4 Study selection

**Figure 1:**
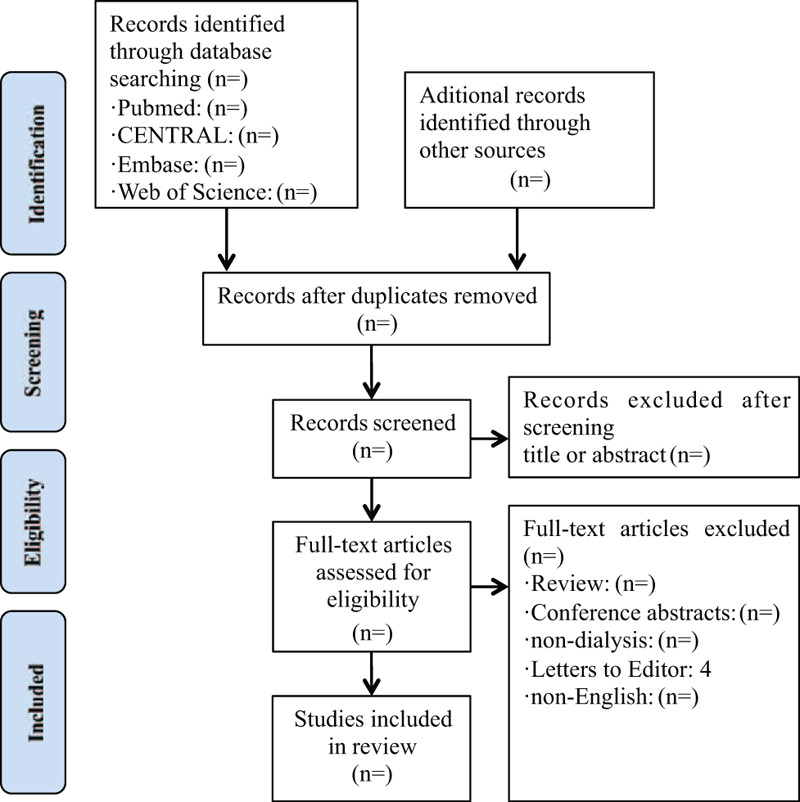
Study flow.

All duplicated studies will be imported into Endnote X7, USA software and excluded before the screening. Two authors will independently scan all the records from title and abstract and all irrelevant literatures will be removed. Then, full manuscripts of all remaining studies will be further identified to check if they meet all inclusion criteria. We will note all excluded citations with specific reasons. If there are any different opinions between 2 authors, we will invite another author for consultation and final decision will be made after discussion. The detail of the study selection will be presented in a PRISMA flow diagram (Fig. 1).

#### 2.4.1 Data extraction

Two authors will independently extract the following associated information from each included trial: first author, time of publication, location, sample size, randomization methods, blinding, concealment, allocation, details of intervention and controls, duration of follow-up, outcome measurement tools, and any other relevant information. A third senior author will help to reconcile any divergences between 2 authors.

#### 2.4.2 Missing data dealing with

If we identify any unclear or missing data, we will contact original authors to obtain them. If we cannot get reply, we will only analyze available data and will discuss its potential affect as limitation.

#### 2.4.3 Quality assessment

Two independent reviewers assessed the methodological quality by using the Newcastle-Ottawa Scale with some modifications to match the needs of this study.^[11,12]^ The quality was evaluated by examining 3 items: selection, comparability, and exposure, with higher scores representing studies of higher quality. The quality of each study was graded as either level 1 (0–5) or level 2 (6–9).^[13]^ This review also assessed the clinical heterogeneity to evaluate whether the trials were similar enough to pool data.

#### 2.4.4 Subgroup analysis

We will preside over subgroup analysis to explore any potential heterogeneity and inconsistency based on the number of levels.

#### 2.4.5 Sensitivity analysis

We will consider running sensitivity analysis to identify the robustness and stability of merged results by excluding studies with high risk of bias.

#### 2.4.6 Reporting bias

If necessary, we will examine the reporting bias using funnel plot and Egger regression test when >10 trials are included.

### 2.5 Data synthesis

We will undertake REvman 5.3, USA software to analyze data and to perform meta-analysis if it is necessary. We will calculate all continuous data using mean difference or standardized mean difference and 95% confidence intervals (CI). As for dichotomous data, we will exert it using risk ratio and 95% CI. The heterogeneity as determined by the Cochran statistics was <0.10 of the chi-squared test. If the *I*^2^ value was >50%, we marked it as a considerable level of heterogeneity; otherwise, we considered it to be a good homogeneity. We also assessed clinical heterogeneity. Statistically and clinically homogeneous studies were pooled using a fixed-effects model; otherwise, a random-effects model was used when the heterogeneity was significant. Additionally, subgroup analysis will be operated to explore any possible reasons for the high heterogeneity. Whenever it is possible, we will conduct meta-analysis if at least 3 eligible criteria are fulfilled. Otherwise, meta-analysis will not be carried out if only 1 or 2 studies meet the inclusion criteria. Under such situation, the findings will be presented in a narrative summary. We will perform narrative synthesis if running meta-analysis is inappropriate due to the high heterogeneity. All narrative descriptions will be carried out based on the Guidance on the Conduct of Narrative Synthesis in Systematic Reviews.

## 3 Discussion

Anterior cervical decompression by discectomy followed by fusion is a widely accepted and safe surgical procedure for the treatment of degenerative cervical spine disease.^[14]^ The primary aim of this technique is to decompress the spinal cord and the affected nerve roots while restoring cervical alignment.

The intervertebral fusion device named Zero-profile is a new kind of cervical fusion system that can be independently applied to single-segment or a multi segment anterior cervical spondylosis.^[15]^ This device has the benefits of both of the cage and the anterior plate. A Zero-profile fusion implant in the intervertebral space after decompression will not be prominent in the vertebral column. Owing to its design, Zero-profile can significantly limit the potential risks of postoperative dysphagia and degeneration of adjacent segments after the internal fixation in anterior cervical fusion surgery. Furthermore, and in particular, it can increase the immediate stability of the treated segments.^[16]^

The results of the present review showed strong statistical evidence for the clinical efficacy of the 2 anterior cage systems in the treatment of symptomatic cervical spondylosis. However, it is important to emphasize that the quality of the evidence is low because of the lack of randomized clinical trials (RCTs); moreover, the included observational studies could have selection bias and most studies had a small sample size, leading to a lack of statistical power.^[17]^

The strength of this systematic review and meta-analysis will include: search a comprehensive range of databases, including Chinese and English databases, more rigorous and detailed concerning quality assessment, and data extraction. In addition, the findings obtained in the present study will provide helpful evidence in clinical practice. Furthermore, it will also help to promote further studies and clarify the direction for the future research.

On the contrary, this study has several potential limitations. There may be a language bias, although there is not language limitation in this study. Moreover, there may be a large heterogeneity, which may bias the results.

## Author contributions

Rui Wang, Wei Teng, and Jinhua Yu have conducted of the protocol and drafting the manuscript. All authors participated in the design of the study. All authors read and approved the final manuscript.

## References

[1] Tracy JA Bartleson JD Cervical spondylotic myelopathy. Neurologist 2010;16:176 87. 20445427 10.1097/NRL.0b013e3181da3a29

[2] Vanek P Bradac O DeLacy P Comparison of 3 fusion techniques in the treatment of the degenerative cervical spine disease. Is stand-alone autograft really the “gold standard?”: prospective study with 2-year follow-up. Spine (Phila Pa 1976) 2012;37:1645 51. 22433506 10.1097/BRS.0b013e31825413fe

[3] Korinth MC Treatment of cervical degenerative disc disease - current status and trends. Zentralbl Neurochir 2008;69:113 24. 18666050 10.1055/s-2008-1081201

[4] Le H Thongtrangan I Kim DH Historical review of cervical arthroplasty. Neurosurg Focus 2004;17:1 3. 10.3171/foc.2004.17.3.1 15636556

[5] Matz PG Ryken TC Groff MW Techniques for anterior cervical decompression for radiculopathy. J Neurosurg Spine 2009;11:183 97. 19769498 10.3171/2009.2.SPINE08721

[6] Gercek E Arlet V Delisle J Subsidence of stand-alone cervical cages in anterior interbody fusion: warning. Eur Spine J 2003;12:513 6. 12827473 10.1007/s00586-003-0539-6 PMC3468003

[7] Schmieder K Wolzik-Grossmann M Pechlivanis I Subsidence of the wing titanium cage after anterior cervical interbody fusion: 2-year follow-up study. J Neurosurg Spine 2006;4:447 53. 16776355 10.3171/spi.2006.4.6.447

[8] Yue WM Brodner W Highland TR Persistent swallowing and voice problems after anterior cervical discectomy and fusion with allograft and plating: a 5- to 11-year follow-up study. Eur Spine J 2005;14:677 82. 15692825 10.1007/s00586-004-0849-3 PMC3489223

[9] Fountas KN Kapsalaki EZ Nikolakakos LG Anterior cervical discectomy and fusion associated complications. Spine (Phila Pa 1976) 2007;32:2310 7. 17906571 10.1097/BRS.0b013e318154c57e

[10] Moher D Liberati A Tetzlaff J Preferred reporting items for systematic reviews and meta-analyses: the PRISMA statement. PLoS Med 2009;6:e1000097. 19621072 10.1371/journal.pmed.1000097 PMC2707599

[11] Taggart DP D’Amico R Altman DG Effect of arterial revascularisation on survival: a systematic review of studies comparing bilateral and single internal mammary arteries. Lancet 2001;358:870 5. 11567701 10.1016/S0140-6736(01)06069-X

[12] Wells G, Shea B, O’Connell D, et al. New Castle-Ottawa Quality Assessment Scale --Cohort Studies. Available at: http://www.ohri.ca/programs/clinical_epidemiology/oxford.asp.

[13] Athanasiou T Al-Ruzzeh S Kumar P Off-pump myocardial revascularization is associated with less incidence of stroke in elderly patients. Ann Thorac Surg 2004;77:745 53. 14759484 10.1016/j.athoracsur.2003.07.002

[14] Liu Y Hou Y Yang L Comparison of 3 reconstructive techniques in the surgical management of multilevel cervical spondylotic myelopathy. Spine (Phila Pa 1976) 2012;37:E1450 8. 22869063 10.1097/BRS.0b013e31826c72b4

[15] Barsa P Suchomel P Factors affecting sagittal malalignment due to cage subsidence in standalone cage assisted anterior cervical fusion. Eur Spine J 2007;16:1395 400. 17221174 10.1007/s00586-006-0284-8 PMC2200763

[16] Lee MJ Bazaz R Furey CG Influence of anterior cervical plate design on Dysphagia: a 2-year prospective longitudinal follow-up study. J Spinal Disord Tech 2005;18:406 9. 16189451 10.1097/01.bsd.0000177211.44960.71

[17] P Oprel P Tuinebreijer WE Patka P Combined anterior-posterior surgery versus posterior surgery for thoracolumbar burst fractures: a systematic review of the literature. Open Orthop J 2010;4:93 100. 21283533 10.2174/1874325001004010093 PMC3031139

